# Probabilistic Modeling of Motion Blur for Time-of-Flight Sensors

**DOI:** 10.3390/s22031182

**Published:** 2022-02-04

**Authors:** Bryan Rodriguez, Xinxiang Zhang, Dinesh Rajan

**Affiliations:** Lyle School of Engineering, Department of Electrical and Computer Engineering, Southern Methodist University, Dallas, TX 75205, USA; brodrigu@mail.smu.edu (B.R.); rajand@lyle.smu.edu (D.R.)

**Keywords:** 3D image processing, depth maps, time-of-flight sensors

## Abstract

Synthetically creating motion blur in two-dimensional (2D) images is a well-understood process and has been used in image processing for developing deblurring systems. There are no well-established techniques for synthetically generating arbitrary motion blur within three-dimensional (3D) images, such as depth maps and point clouds since their behavior is not as well understood. As a prerequisite, we have previously developed a method for generating synthetic motion blur in a plane that is parallel to the sensor detector plane. In this work, as a major extension, we generalize our previously developed framework for synthetically generating linear and radial motion blur along planes that are at arbitrary angles with respect to the sensor detector plane. Our framework accurately captures the behavior of the real motion blur that is encountered using a Time-of-Flight (ToF) sensor. This work uses a probabilistic model that predicts the location of invalid pixels that are typically present within depth maps that contain real motion blur. More specifically, the probabilistic model considers different angles of motion paths and the velocity of an object with respect to the image plane of a ToF sensor. Extensive experimental results are shown that demonstrate how our framework can be applied to synthetically create radial, linear, and combined radial-linear motion blur. We quantify the accuracy of the synthetic generation method by comparing the resulting synthetic depth map to the experimentally captured depth map with motion. Our results indicate that our framework achieves an average Boundary F1 (BF) score of 0.7192 for invalid pixels for synthetic radial motion blur, an average BF score of 0.8778 for synthetic linear motion blur, and an average BF score of 0.62 for synthetic combined radial-linear motion blur.

## 1. Introduction

The ability to synthetically create motion blur in 2D and depth images is useful for a wide range of applications. In the recent decade, increasingly research of developing more effective and adaptive deblurring techniques has received significant attentions in applications such as 3D scanning [1,2], structural health monitoring [3,4,5,6], drone positioning [7,8,9], robotics [10,11,12], road surveillances [13,14,15], and logistics [16,17,18]. In such applications, there could be relative motion between the sensor and objects within its field of view (FOV). The relative movement between the sensor and other objects result in motion blur which increases the number of blurring pixels and flying pixels that are present in a 2D image or a depth map and distorts the appearance of the objects [19]. Several types of motion blur exist such as radial motion, linear motion, out-of-focus blur, or a combination of blur types. Synthetically creating motion blur in 2D images is a well-understood process [20,21]. In traditional 2D images, motion blur appears as a softening of edges within a 2D image along a motion path. Previous works have developed different models and techniques for creating motion blur in 2D images [22,23,24]. However, the motion blur in depth maps is distinct from 2D images because of the presence of invalid pixel values and flying pixels which do not exist in 2D image motion blur. Other previous works primarily focus on different techniques for deblurring 2D images. These deblurring techniques include learning-based approaches [25,26,27] and blind approaches [28,29,30]. Motion blur in a ToF sensor is a function of the integration time of the ToF sensor and the relative motion between the ToF sensor and an object [19,31,32,33,34]. Invalid pixels from motion blur typically appear when a depth transition occurs during the integration time while a depth is being measured. The depth transition causes an ambiguity in the depth measurement which results in an invalid pixel. The locations of invalid pixels are scene-specific, which makes capturing 3D images of motion blur in a real-world environment challenging because the motion path and the speed of objects are not always easily controllable. This challenge makes using non-probabilistic approaches or machine learning approaches that require large training data sets less feasible.

In other previous works, motion blur in depth maps has been observed as a baseline for comparing different 3D sensing technologies or for evaluating the performance of various deblurring algorithms [19,35]. These works typically focus on how to minimize the effects of motion blur and they do not provide any insight about how to synthetically create motion blur. For example, in reference [35], radial motion blur is captured using both a Kinect v1 sensor that uses structured light and a Kinect v2 sensor that uses ToF. For each Kinect sensor, a depth map is generated for a flat fan blade, a Siemens Star, while it is static and while it is rotating and the distortion between the static and rotating depth maps is computed. The focus of reference [35] is simply to compare the amount of distortion that is present in the depth maps generated by the different Kinect sensors.

In our previous work [36], we introduced a probabilistic model to predict the location of invalid pixels, also referred to as zero-value pixels, that are present when motion blur occurs. The model in [36] can only be applied to create radial and linear motion blur for motion that is parallel to the image plane of a ToF sensor and does not work for motion that is not parallel to the image plane of the ToF sensor. Reference [36] is also limited to only synthetically creating either radial or linear motion blurs that are independent of each other and is unable to create a combined radial-linear motion blur.

This work presents a framework for synthetically generating motion blur within a depth map that mimics the real motion blur that is observed in ToF sensor data. Our framework can be applied to radial, linear, and combined radial-linear motion blur and takes into account the speed, motion type, and motion path of the object. Using our framework, a synthetic radial, linear, or combined radial-linear motion blur can be applied to depth maps of static objects. If desired, the blurred depth map can then also be converted into a point cloud that will include the applied motion blur. The ability to synthetically generate motion blur in 3D images enables future works to create test benches for evaluating algorithms for deblurring this motion blur. To the best of our knowledge, there are no well-established techniques for synthetically generating motion blur within 3D images such as depth maps and point clouds.

This work contributes to the state of the art by (1) developing a framework for synthetically generating motion blur in depth maps that mimics the appearance and behavior of real motion blur that can be observed using a ToF sensor; (2) developing the probabilistic model to predict the locations of invalid pixels to synthetically generate combined radial-linear motion blur; and (3) conducting extensive experiments to verify the performance of our framework for generating synthetic motion blur in depth maps. Our results indicate that our framework is able to achieve an average BF score of 0.7192 for invalid pixels for synthetic radial motion blur, an average BF score of 0.8778 for synthetic linear motion blur, and an average BF score of 0.62 for synthetic combined radial-linear motion blur. Our results also indicate that our framework is able to achieve a BF score between 0.7802 and 0.7873 for radial motion blur and a BF score between 0.8498 and 0.8954 for linear motion blur when the object plane of motion is rotated between zero and thirty degrees with respect to the image plane of a ToF sensor.

This work uses a Kinect v2 ToF sensor for experiments due to its popularity and widespread use in research and engineering applications. In addition, the Kinect v2 also provides the most control among commercial ToF sensors by allowing us to disable its internal Bilateral filter and Edge-aware filter to access the raw data from the ToF sensor. However, we also demonstrate that our framework for synthetically generating motion blur within a depth map can generally be applied to other types of ToF sensors.

This paper is organized as follows. Section 2 discusses our methodology for synthetically generating motion blur within a depth map. Section 3 describes our experimental setup and results. Section 4 provides concluding remarks.

## 2. Methodology

**Figure 1:**
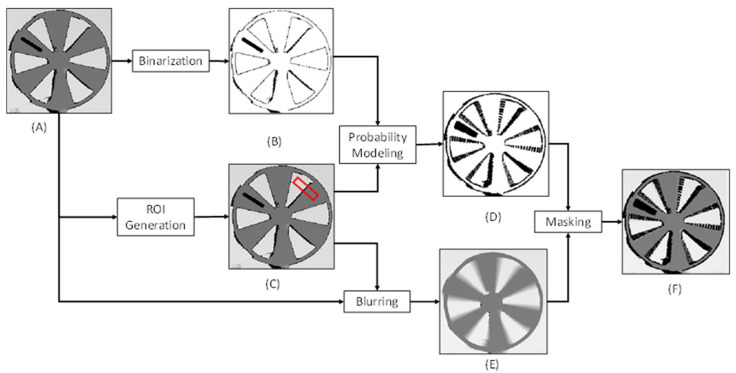
Process for generating synthetic motion blur. (**A**) Initial depth map from ToF sensor; (**B**) Binary depth map; (**C**) Depth map with an example of an ROI for a pixel shown in red. The ROI has been enlarged for visualization purposes; (**D**) Binary depth map after updating the binary depth with the predicted zero value pixels. The zero-value pixels are shown in black and the non-zero value pixels are shown in white; (**E**) Depth map after applying the blur filter; (**F**) Blurred depth map after applying predicted zero value pixels. For visualization purposes, (**A**,**C**,**E**,**F**) are shown within normalized values in the uint8 range.

Motion blur appears in depth maps as an increase in the number of zero-value pixels (i.e., invalid pixels) and flying pixels that result in near depth discontinuities within a scene [19]. In general, ToF sensors determine depth values based on the amount of time it takes for infrared (IR) light that is emitted from the ToF sensor to return to the ToF sensor, after reflecting off a surface within a scene. As an object moves with respect to the ToF sensor, the IR light that is reflected near the edges of the object may result in erroneous depth values that do not accurately represent the surface of the object. The increase in the number of zero-value pixels and flying pixels results in fewer pixels on the surface of the object which tends to distort the appearance of the object within depth maps. For example, the distorted appearance may cause the surface area of an object to reduce and the size of openings to increase. In this work, we propose an approach for synthetically applying motion blur to the depth map of a static object. After synthetically applying the motion blur to the depth map, the resulting depth map mimics the appearance and behavior of a depth map that would be observed if the object were moving. Figure 1 provides an overview of our methodology, and the frequently used notations are provided in Table 1.

The key steps to the synthetic motion blur generation methodology are as follows. (1) A static ToF sensor is used to capture the depth map of a static object. We then perform a binarization on the depth map to generate a binary depth map by setting the non-zero values from the depth map to a value of one and the zero values, which correspond with invalid pixels, to a value of zero. (2) We then define a region-of-interest (ROI) for each pixel in the depth map and the binary mask. (3) After defining the ROIs, we then blur the depth map. This blurring process involves first performing interpolation using the previously determined ROIs to assign values to the pixels in the depth map that have a value of zero. After interpolation, we then apply a spatial blur filter to the depth-map that averages the pixel values of each pixel based on the pixel values of neighboring pixels within each of their respective ROIs. (4) We then use probabilistic modeling to predict the locations of new zero-value pixels within the binary depth map. The probabilistic model predicts whether a pixel location within the depth map should have a zero value after the spatial blur filter is applied. The result of this process is an updated binary depth map that includes the additional predicted zero value pixels. (5) We then mask the blurred depth map using the updated binary depth map to add the predicted zeros to the blurred depth map. This process is discussed in more detail below.

### 2.1. ROI Generation

**Figure 2:**
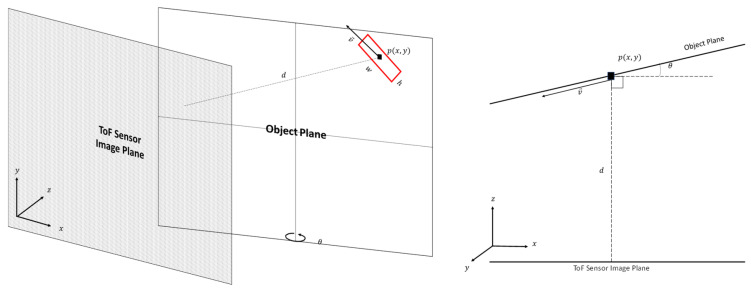
Perspective view of a relationship of between a ToF sensor image plane and motion of an object in an object plane. For simplicity, the object's movement is along a single plane (**left**). Top view of the relationship between the ToF sensor image plane and the movement of the object in the object plane (**right**).

Figure 2 shows a perspective view of a relationship of between a ToF sensor image plane and motion of an object in an object plane. In our work, the ToF sensor is positioned at a distance, *d*, from an object. For simplicity, we consider the motion of a single object in the FOV. However, our methodology can be easily extended to multiple object motion. Let the object motion plane have a rotation angle, 𝜃, with respect to the ToF sensor image plane. For simplicity, this study uses a single rotation angle with respect to the y-axis of the ToF sensor image plane. However, the principles used in this work can be applied to the other axes of the ToF sensor image plane.

As the object moves, an initial depth map, ***I*** ∈ ℝ^M×N^, is obtained from the ToF sensor where ***I*** is a 2D matrix of pixels with the dimensions M × N. Each given pixel within the depth map is identified as *p*(*x*, *y*). After obtaining the depth map, an ROI (e.g., a bounding box) is defined for each pixel within the initial depth map based on velocity of the object, $\vec{v}$. In our framework, the ROI represents neighboring pixels {*n*_0_, *n*_1_, …, *n_l_*}, where {*n*_0_, *n*_1_, …, *n_l_*} ∈ ***I***, that influence the value of a given pixel during the motion blurring process.

In general, the size of the ROI is proportional to the speed of the object, and the orientation of the ROI is associated with the motion type for the object. In this work, we consider linear motion and radial motion since these types of motion blur are the most applicable to a variety of applications. The ROI dimensions for radial motion and/or linear motion can be calculated using Equation (1) and Equation (2). For each pixel *p*(*x*, *y*) we determine its radial velocity as $\vec{v_{R}}$, linear velocity as $\vec{v_{L}}$, and the angle between $\vec{v_{R}}\;$and $\vec{v_{L}}$ as *γ*. In this way, the dimensions for an ROI are calculated as follows.

(1) $$w=s\left(x,\;y\right)\cdot \left(\left|\vec{v_{R}}\right|+\left|\vec{v_{L}}\right|\cdot \cos\gamma \right)$$

where *w* is the ROI dimension tangential to the velocity of a pixel and *s*(*x*, *y*) is a perspective distortion scaling function.

(2) $$h=s\left(x,\;y\right)\cdot \left(H+\left|\vec{v_{L}}\right|\cdot \sin\gamma \right)$$

where *h* is the ROI dimension orthogonal to the velocity of the pixel and *H* is a user-defined constant height value. For simplicity, this study assumes that both radial motion and linear motion are in the same object plane and that any velocity components that are orthogonal to the object plane are negligible. Future work may extend Equations (1) and (2) to account for velocity components that are orthogonal to the object plane. In the following sub-sections, we discuss how $\vec{v_{R}}$, and $\vec{v_{L}}$ are determined for an object. The perspective distortion scaling function, *s*(*x*, *y*), is described in Section 2.1.3.

#### 2.1.1. Radial Velocity

**Figure 3:**
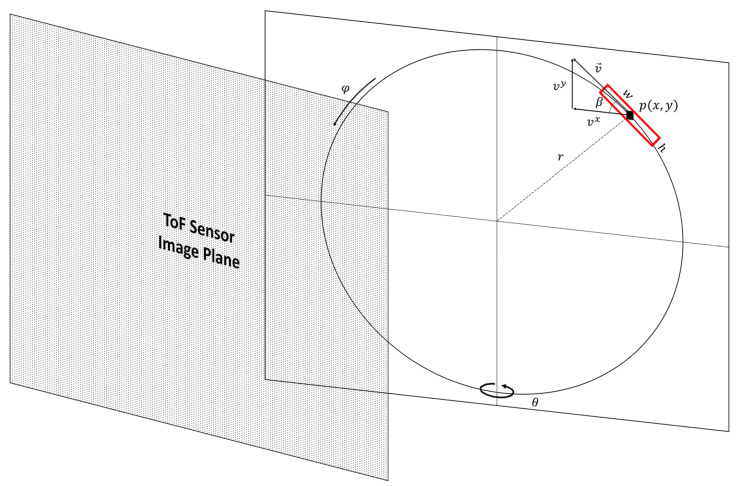
Radial motion with respect to the image plane of the ToF sensor.

Figure 3 illustrates a planar object with radial motion. For an object with radial motion, the velocity of the object is along a path that is tangential to the direction of motion and its magnitude can be expressed as follows:

(3) $$\left|\vec{v}\right|=\alpha \cdot r\left|\vec{\varphi }\right|$$

where *α* is a scaling factor for converting radial motion to the pixel domain, *r* is the radius of radial motion, and $\vec{\varphi }$ is the angular velocity of the object. In our experiments, the angular velocity of the object is constant and depends on the speed of the motor that is driving the radial motion. The relationship between the velocity, $\vec{v}$, of a pixel when the motion path of the pixel is not parallel with the image plane of the ToF sensor and the velocity, $\vec{v_{0}}$, of the pixel when the motion path of the pixel is parallel with the image plane of the ToF sensor is given as:

(4) $$\left|v^{x}\right|=\left|v_{0}^{x}\right|\;\cdot \cos\theta$$

where

$$\left|\vec{v}\right|=\sqrt{{\left|v^{X}\right|}^{2}+{\left|v^{Y}\right|}^{2}}\;\left|\vec{v_{0}}\right|=\sqrt{{\left|v_{0}^{X}\right|}^{2}+{\left|v_{0}^{Y}\right|}^{2}}\;\left|v^{Y}\right|=\left|v_{0}^{Y}\right|\;\;\beta_{0}=\tan^{-1}\frac{\left|v_{0}^{Y}\right|}{\left|v_{0}^{X}\right|}$$

In Equation (4), |$v^{X}$| and |$v^{Y}$| are the magnitudes of the velocity component in the x-direction and y-direction, respectively, when the motion path of the pixel is not parallel with the image plane of the ToF sensor, |$v_{0}^{x}$| and |$v_{0}^{Y}$| are magnitudes of the velocity component in the x-direction and y-direction, respectively, when the motion path of the pixel is parallel with the image plane of the ToF sensor, *β*_0_ is the rotation angle of the pixel when the motion path of the pixel is parallel with the image plane of the ToF sensor, and 𝜃 is the rotation angle between the image plane of the ToF sensor and the motion of the object. Using this relationship, we can then determine the velocity for a pixel with radial motion by solving the following:

(5) $${\left|\vec{v}\right|}^{2}-{\left|\vec{v_{0}}\right|}^{2}={\left(\;\sqrt{{\left|v^{X}\right|}^{2}+{\left|v^{Y}\right|}^{2}}\right)}^{2}-{\left(\sqrt{{\left|v_{0}^{X}\right|}^{2}+{\left|v_{0}^{Y}\right|}^{2}}\right)}^{2}$$

where

$${\left|\vec{v}\right|}^{2}={\left|v_{0}^{X}\right|}^{2}\cdot \;\cos^{2}\theta +{\left|v_{0}^{Y}\right|}^{2}\;{\left|\vec{v_{0}}\right|}^{2}={\left|v_{0}^{X}\right|}^{2}+{\left|v_{0}^{Y}\right|}^{2}\;{\left|v_{0}^{Y}\right|}^{2}={\left|\vec{v_{0}}\right|}^{2}+{\left|v_{0}^{X}\right|}^{2}\;{\left|v_{0}^{X}\right|}^{2}={\left|v_{0}\right|}^{2}\cdot \sin\beta_{0}$$

Equation (6) is the result from solving Equation (5) which can be used in Equation (1) to determine the dimension tangential to the velocity of a pixel for an ROI.

(6) $$\left|\vec{v}\right|=\sqrt{{\left|\vec{v_{0}}\right|}^{2}\cdot \;\left(\sin^{2}\beta_{0}\cdot \cos^{2}\theta -\sin^{2}\beta_{0}+1\right)}$$

After determining the dimensions of the ROI, we can then determine the rotation angle, *β*, of the ROI as:

(7) $$\beta =\tan^{-1}\left(\frac{\left|v^{Y}\right|}{\left|v^{X}\right|}\right)$$

After determining the dimensions and orientation of the ROI, the pixel is then centered within the ROI.

#### 2.1.2. Linear Velocity

**Figure 4:**
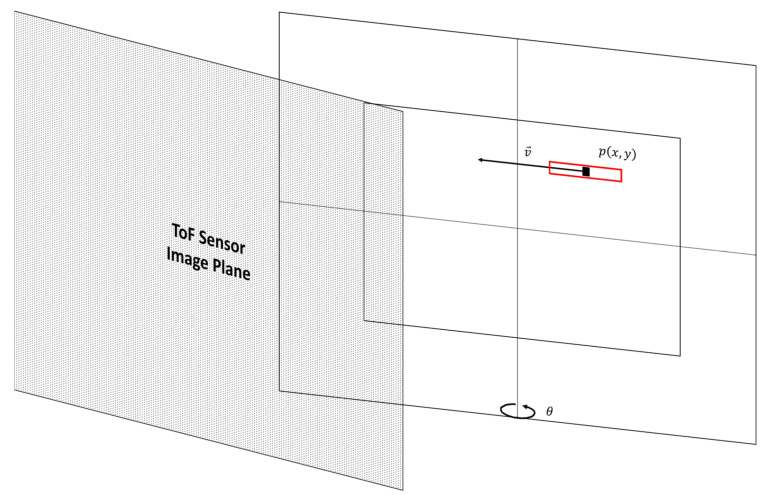
Linear motion with respect to the image plane of the ToF sensor.

Figure 4 illustrates a planar object with linear motion. For an object with linear motion, the velocity of the object is along a path that is tangential to the direction of motion and its magnitude is equal to the product of a scaling factor, *α*, and the linear velocity, |$\vec{\mu }$|, of the object. The scaling factor *α* is used to convert the linear velocity from real-world units (such as meters per second) to the pixel units (pixels per second). In our experiments, the linear velocity, |$\vec{\mu }$|, of the object is constant and depends on the speed of the motor that is driving the linear motion and is given by:

(8) $$\left|\vec{v}\right|=\left|\vec{v_{0}}\right|\cdot \cos\theta$$

where

$$\left|\vec{v_{0}}\right|=\alpha \cdot \left|\vec{\mu }\right|$$

In this case, the rotation angle, *β*, of ROI is approximated to be along a path that is parallel to the direction of motion (i.e., *β* = 0).

#### 2.1.3. Perspective Distortion Function

**Figure 5:**
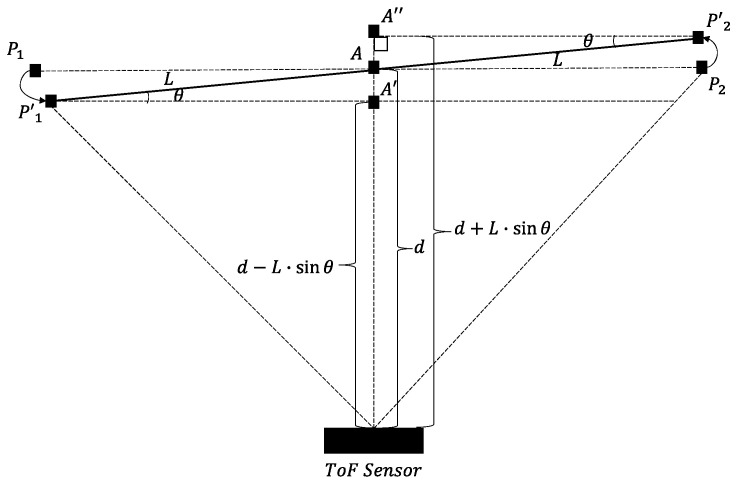
Top view of an object rotated with respect to the image plane of the ToF sensor.

As the rotation angle, 𝜃, becomes non-zero, perspective distortion begins to appear in the depth maps which causes portions of the object that are closer to the ToF sensor to occupy more pixels in the depth map than portions of the object that are further away from the ToF sensor. Figure 5 illustrates a top view of an object that is rotated with respect to the image plane of a ToF sensor. In this study, only horizontal distortions are considered. As shown in Figure 5, the object is orientated such that *d* is the distance between the ToF sensor and a midpoint, A, of the object, L is half of the length of the object in real-world units, P_1_ and P_2_ are points on opposite ends of the object when there is no rotation between the object and the image plane of the ToF sensor, P’_1_ and P’_2_ are points on opposite ends of the object when there is a rotation between the object and the image plane of the ToF sensor, A’ is the midpoint of the object corresponding with the point P’_1_, and A’’ is the midpoint of the object corresponding with the point P’_2_. As denoted earlier, N is the width of the depth map in pixels.

To account for this perspective distortion, our framework uses a linear approximation using similar perspective triangles [37]. In this way, the estimated perspective distortion scaling function between a minimum and a maximum distortion factor can then be applied to Equations (1) and (2) based on the distance between a particular pixel *p*(*x*, *y*) on the object and the ToF sensor. Thus, the perspective distortion scaling function, *s*(*x*, *y*), is a function of the pixel location, *p*(*x*, *y*) and is given by:

(9) $$s\left(x,y\right)=\left(x-N\right)\cdot \left(\frac{s_{max}-s_{min}}{-N}\right)+s_{min}$$

where the maximum scaling factor, *s_max_*, and the minimum scaling factor, *s_min_*, are given respectively, by:

(10) $$S_{max}=\frac{d}{d-L\cdot \sin\theta }$$

(11) $$S_{min}=\frac{d}{d+L\cdot \sin\theta }$$

While these equations provide for a first-order approximation of the perspective distortion, a more comprehensive methodology can be used in future works.

### 2.2. Blurring the Depth Map

The first step in the blurring process is to perform a bilinear interpolation on the original depth map. The depth map that is obtained from the ToF sensor includes a combination of zero-value and non-zero value pixels. A zero-value pixel is an invalid pixel where the ToF sensor was unable to determine a depth value. A non-zero pixel is a pixel where the ToF sensor was able to determine depth value. We perform the bilinear interpolation process to assign the zero-value pixels a depth value based on their neighboring pixels within an ROI as defined in Section 2.1. This interpolation process allows the subsequent blurring process to be applied more accurately to the depth map. After interpolation, we then apply a spatial filter to the depth map to simulate the appearance of a motion blur. In this work, we apply a blur filter to generate a blurred depth map, ***I****_f_*, with the same dimensions as the input depth map ***I***. For combined radial-linear motion blur, the input is the ROIs for each pixel that was defined in Section 2.1. For radial motion blur, the input is the ROIs for each given pixel that was defined in Section 2.1.1. For linear motion blur, the input is the ROIs for each pixel that was defined in Section 2.1.2.

### 2.3. Probability Modeling

In this section, we discuss our new probabilistic model that predicts the location of zero-value pixels in the motion-blurred depth map based on values of neighboring pixels. The new probabilistic model improves the probabilistic model from [36] which could only predict the location of zero-value pixels when the motion of an object is parallel to the image plane of a ToF sensor and was limited to either radial motion blur or linear motion blur only. Our new probabilistic model is capable of predicting the location of zero-value pixels when the motion of an object is not parallel to the image plane of a ToF sensor and works for combined radial-linear motion blur.

When real motion blur occurs in the depth map from a ToF sensor, there is an increase in the number of zero-value pixels that are present near discontinuities and edges in the depth map. As the speed of the object increases, the number of zero-value pixels also increases. To predict the locations of new zero-value pixels our probabilistic model uses the same three assumptions as used in reference [36]. (1) The number of neighboring pixels that are in the bounding box for predicting the final state of a pixel is proportional to the speed of the object. (2) For each pixel, the neighboring pixels in the bounding box with the same state (i.e., 0 or 1) will contribute more to the final state of the pixel. The final state of the pixel is also more likely to be affected by neighboring pixels with a state of zero because zero-value pixels tend to appear in clusters. (3) The pixels with an initial state of zero are likely to remain in state zero. In Section 3.2, we provide experimental results that confirm the assumptions used in the probabilistic model.

To use the probabilistic model, we first binarize the original depth map into a binary depth map, ***I****_b_*, as follows.

(12) $$I_{b}\left(x,\;y\right)=\left\{\begin{array}{cc} \;1,\; & I\left(x,\;y\right)\neq 0\\ \;0,\; & I\left(x,\;y\right)=0 \end{array}\right.$$

where ***I****_b_*(*x*, *y*) is the binary value of ***I****_b_* at pixel *p*(*x*, *y*) and ***I***(*x*, *y*) is the sensed value of ***I*** at pixel *p*(*x*, *y*). We then use the probabilistic model to predict whether a pixel should be a zero-value pixel in the motion-blurred depth map based on its neighboring pixels. For each selected pixel, we use the ROIs defined in Section 2.1 based on the type of motion blur that is being applied. In general, the ROI dimension tangential for a given pixel is proportional to the speed of the object. Thus, a larger ROI tangential dimension is used to simulate faster-moving objects. The orientation (i.e., the rotation angle) of the ROI is configured to be along the motion path of the object. When the direction of motion for the object is known, the ROI can be positioned to include neighboring pixels that precede the pixel in the direction of motion. When the direction of motion for the object is unknown, the ROI can be defined to include neighboring pixels that both precede and follow the pixel. Clearly, the accuracy of the blurring in this case would be lower and should be investigated in future work. The ROI orthogonal dimension does not change based on the direction of motion. Instead, the ROI is oriented to be tangential to the direction of motion. As an example, for a given pixel *p*(*x*, *y*), we use the defined bounding box to identify a group of neighboring pixels, {*n*_0_, *n*_1_, …, *n_l_*}, using the process described in Section 2.1.

After identifying the neighboring pixels for the given pixel, we then apply our probabilistic model to the identified neighboring pixels. We first use a potential function that captures the interaction between the state of the neighboring pixel and the state of the pixel as:

(13) $$\varepsilon \left(s_{p},\;s_{n_{i}}\right)=\left\{\begin{array}{c} \left(1-\left|s_{n_{i}}-s_{p}\right|\right)p_{x}+\left|s_{n_{i}}-s_{p}\right|(1-p_{x}),\;s_{p}=0\\ \left(1-\left|s_{n_{i}}-s_{p}\right|\right)(1-p_{x})+\left|s_{n_{i}}-s_{p}\right|p_{x},\;s_{p}=1 \end{array}\right.$$

where *ε*(*s_p_*, *s_ni_*) is the potential function of the *i*th neighboring pixel, *s_ni_* is the state of the *i*th neighboring pixel, and *s_p_* is the state of the pixel having a value of one or zero. The parameter *p_x_* is a user-defined probability weight parameter that can be set to any value between zero and one. We also consider the prior knowledge of the pixel by determining an initial probability, *p_p_*, for the pixel having a value of zero. The probability *p_p_* is determined as:

(14) $$p_{p}=\left\{\begin{array}{c} p_{y},\;s_{p}=0\\ 1-p_{y},\;s_{p}=1 \end{array}\right.$$

where *p_p_* is the initial probability of a pixel having a value of zero and *s_p_* is the state of the pixel having a value of one or zero. The parameter *p_y_* is a user-defined probability weight parameter that can be set to any value between zero and one. To handle the uncertainty of a pixel that is assigned to a zero value in decision-making process [38,39], this process ensures that a higher probability weight is used when a neighboring pixel has the same state as the pixel and when the pixel has an initial state of zero. We then predict a probability for each pixel based on the modeled potential function for each of its neighboring pixels and the initial probability for the pixel. The predicted probability, ${\tilde{p}}_{p}$, of the pixel having a value of zero is calculated as:

(15) $${\tilde{p}}_{p}=\frac{1}{l}\cdot \overset{l}{\underset{i=0}{\sum }}\frac{\varepsilon \left(s_{p},\;s_{n_{i}}\right)*p_{p}}{\rho }$$

where *ρ* is the normalization factor. We then assign the probability to the pixel that corresponds with the likelihood that the pixel will be a zero-value pixel in the blurred depth map. This process is repeated to assign probabilities for all of the given pixels within the region-of-interest for the object. We then apply a threshold to identify pixels that will convert to zero-value pixels in the blurred depth map. The predicted state ${\hat{s}}_{p}$ for the pixel having a value of one or zero in the binary depth map is given by:

(16) $${\hat{s}}_{p}=\left\{\begin{array}{c} 0,\;{\tilde{p}}_{p}>t\\ 1,\;{\tilde{p}}_{p}\leq t \end{array}\right.$$

where ${\tilde{p}}_{p}$ is the predicted probability for the pixel having a value of zero, and *t* is a user-defined threshold value that can be set to any suitable value. Note that the selection of *p_x_*, *p_y_*, and *t* used in this work were determined based on a separate validation set. Once the zero-value pixels are identified, the blurred depth map described in Section 2.2 is updated by applying a mask to the blurred depth map to add the predicted zero-value pixels from the probabilistic model as follows.

(17) $$I_{\;f}\left(x,\;y\right)=\left\{\begin{array}{cc} \;0, & I_{\;b}\left(x,\;y\right)=0\\ I_{\;f}\left(x,\;y\right), & I_{\;b}\left(x,\;y\right)=1 \end{array}\right.$$

## 3. Experiments

### 3.1. Hardware Configuration

In our experiments, we use a Kinect v2 sensor [35] to generate depth maps at a resolution of 512 × 424 pixels and a framerate of 30 frames per second [40,41]. We used OpenKinect libraries [42] to capture the depth information and MATLAB 2020 [43] to create the synthetic motion blur. In our experiments, we disabled both the Bilateral filter and the Edge-aware filter for the Kinect v2 while capturing depth information, to ensure raw depth information is captured [44].

**Figure 6:**
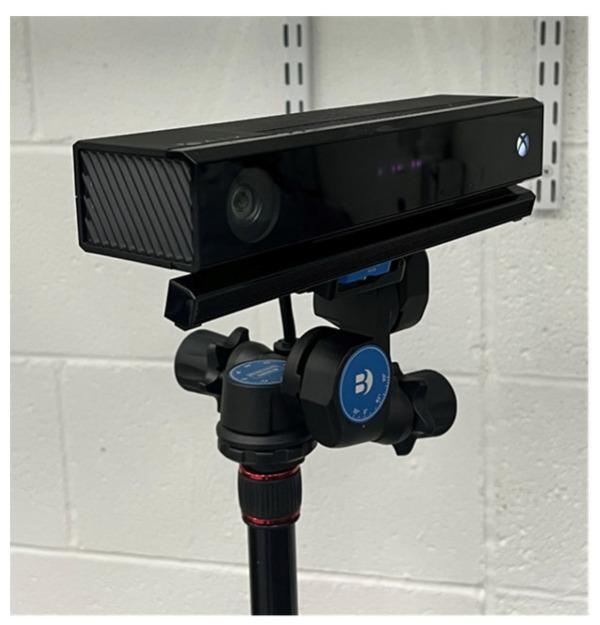
Kinect v2 ToF sensor mounted to a tripod using a 3-way geared head.

The ToF sensor is mounted to a tripod using a Benro 3-way geared head [45] which allows for precise control of the yaw, pitch, and roll of the ToF sensor. We used a dual-axis digital protractor [46] to ensure the ToF sensor is level with the ground plane of our object. The configuration of our ToF sensor is shown in Figure 6.

**Figure 7:**
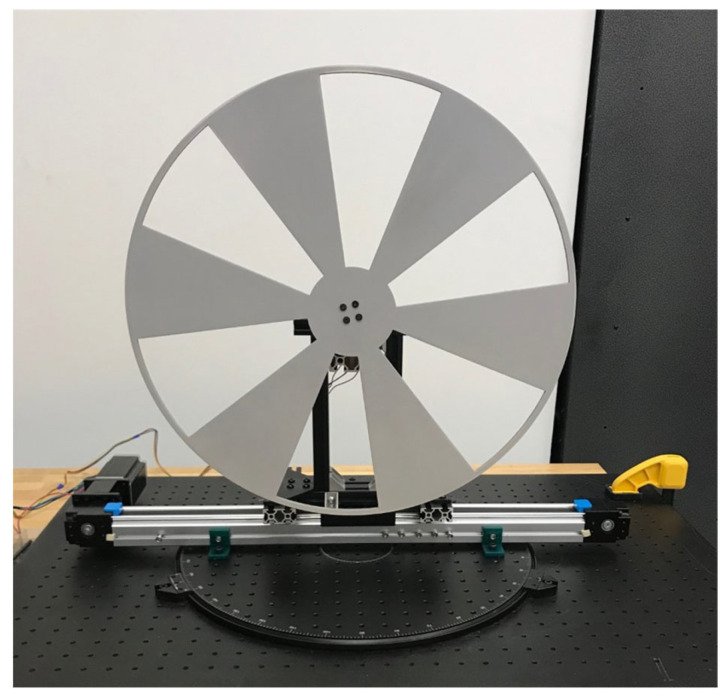
Radial motion device with a Siemens Star mounted to a rotating optical breadboard.

For the experiments with radial motion blur, we used a custom radial motion device that is mounted on an optical table. A rotating optical breadboard [47] is installed onto the optical table surface which allows the radial motion device to be positioned at different rotation angles with respect to the image plane of the ToF sensor. The radial motion device includes a 381 mm diameter Siemens Star with six flat fan blades. The Siemens Star was laser cut from 1/8” acrylic plexiglass. The Siemens Star is attached to a 2.1 A high-torque Nema 17 stepper motor [48] that is controlled using a TB6600 stepper motor driver [49] and an Arduino Uno [50]. We collected data while rotating the Siemens star at 60–135 RPM. Our radial motion device is shown in Figure 7.

**Figure 8:**
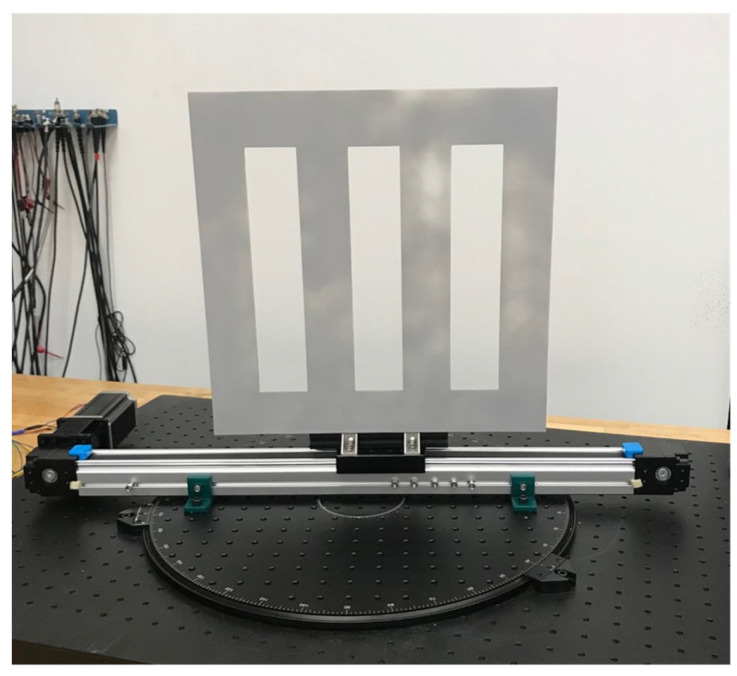
Linear motion device with a target having vertical openings mounted to a rotating optical breadboard.

For the experiments with linear motion blur, we used a custom linear motion device. The linear motion device was also mounted onto the rotating optical breadboard to allow the linear motion device to be positioned at different rotation angles with respect to the image plane of the ToF sensor. The linear motion device includes a target with three vertical openings. The target was laser cut from 1/8” acrylic plexiglass. The dimensions of the target are 381 mm by 381 mm. The dimensions of each vertical opening are 76 mm by 254 mm. The target is mounted to a 500 mm belt drive linear guide rail and is moved along the linear guide rail using a 4.2 A Nema 23 stepper motor [51] that is controlled using a DM542T stepper motor driver [52] and an Arduino Uno [50]. We collected data moving the target at the maximum speed of our linear motion device which is 3.3647 m/s. Our linear motion device is shown in Figure 8.

For the experiments with combined radial-linear motion blur, the radial motion device was mounted to the linear guide rail in place of the target with vertical openings. In this configuration, both the radial motion motor and the linear motion motor are operated simultaneously to create a combination of radial motion blue and linear motion blur. In the combined radial-linear motion blur experiments, the target is configured parallel with the image plane of the ToF sensor. We collected data while rotating the Siemens Star at its maximum speed, 135 RPM, and moving the Siemens Star linearly at the maximum speed of our linear motion device, 3.3647 m/s. This configuration tested our model for the worst case of a motion blur where both the radial motion and the linear motion are at their maximum speeds. The proposed method is still applicable when the target moves in a plane that is not parallel to the ToF sensor plane. The user-defined constant height value, *H*, in the ROI orthogonal dimension is set to a value of one, two, and three pixels, the probability weight parameter *p_x_* is set to a value of 0.9, the probability weight parameter *p_y_* is set to a value of 0.6, and the threshold value *t* is set to a value of 0.05 in all of our experiments.

### 3.2. Verification Results for the Probabilistic Model Assumptions

Table 2 and Table 3 show the results from our experiments to verify the assumptions used for the probabilistic model defined in Section 2.3. Table 2 shows the ratio of zero-value pixels in depth maps with real radial motion blur as velocity and the rotation angle between the Siemens Star and the image plane of the ToF sensor increase. Our results show that the ratio of zero-value pixels in a depth map with real radial motion blur increases as the speed of the object increases. Table 3 shows the percentage of zero-value pixels from a static depth map of the Siemens Star that are still present in a depth map of the Siemens Star with real radial motion blur. Our results show that on average 74.96% of the zero-value pixels from a static depth map will remain a zero-value pixel in a depth map with real radial motion blur. Similar results were observed for an object with linear motion blur.

### 3.3. Performance Metrics

To evaluate the performance of the zero-value pixel predictions from the probabilistic model described in Section 2.3, we use the Boundary F1 (BF) score which is commonly used in a variety of predictive applications including image processing [53]. The BF score provides a metric that measures how well the zero values pixels in the ground truth (i.e., a depth map with real motion blur) match the predicted zero value pixels in the binary depth map after performing our probabilistic modeling. In our experiments, we used a BF score with an error tolerance of 2 pixels.

To evaluate the performance of non-zero value pixels from Section 2.2 after updating the blurred depth map with the predicted zero-value pixels, we use a root-mean-square error (RMSE) between the synthetic motion blur depth map and the real motion blur depth map and an RMSE ratio (RMSE-R) between their difference and the real motion blur depth map. These performance metrics are used because comparing a depth map with synthetic motion blur to a depth map with real motion blur is challenging since the position of objects may be different in both depth maps. Hence, traditional direct comparisons between two depth maps cannot be made without additional considerations to ensure that the position of objects within the depth maps is the same.

Our process for evaluating synthetic motion blur performance involves first capturing a series of depth maps of a static object at different positions that would occur when the object is in motion. We then generate a depth map of the object in motion with real motion blur. The depth maps of the static object are then compared to the depth map with the real motion blur to identify the closest match based on the position of the object. After identifying the depth map of the static object that best matches the depth map of the object in motion, we then apply our synthetic motion blur to the depth map. The depth maps can then be directly compared to quantify the similarity between the two depth maps. This process enables a direct comparison between a depth map with synthetic motion blur and a depth map with real motion blur since we are able to align the position of an object before making the comparison. The ability to compare a depth map with synthetic motion blur and a depth map with real motion blur enables future works to evaluate the performance of other motion blur algorithms.

### 3.4. Radial Motion Blur Experiments

**Figure 9:**
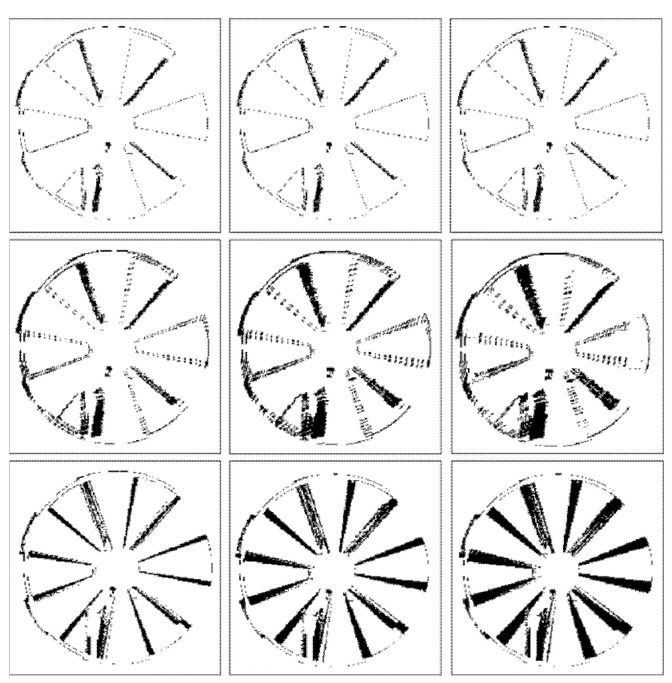
Siemens Star that is parallel to the image plane of the ToF sensor without motion (**top**), with synthetic motion blur (**middle**), with real motion blur (**bottom**) at 60 RPM (**left**), 100 RPM (**center**), and 135 RPM (**right**).

**Figure 10:**
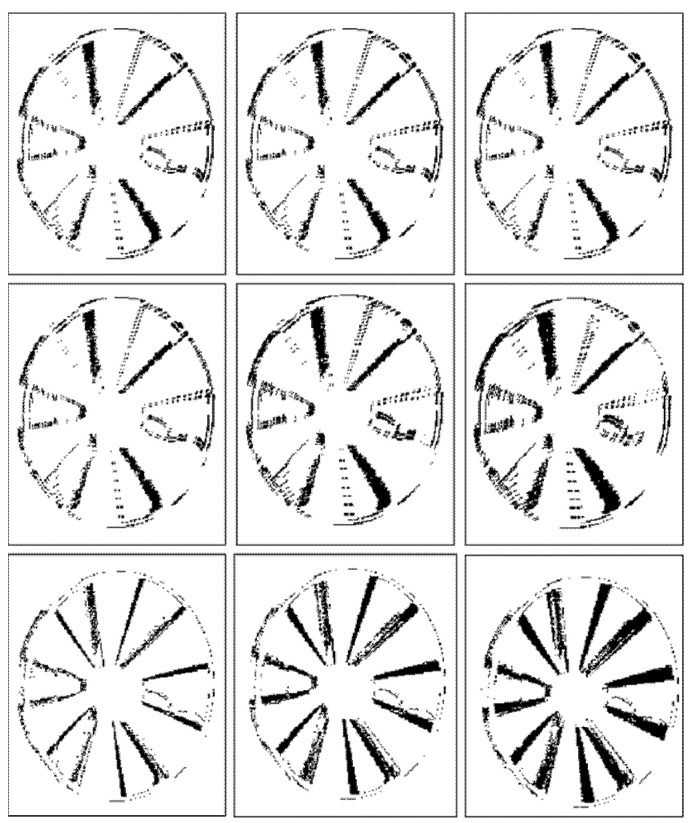
Siemens Star with a 30° rotation from the image plane of the ToF sensor without motion (**top**), with synthetic motion blur (**middle**), with real motion blur (**bottom**) at 60 RPM (**left**), 100 RPM (**center**), and 135 RPM (**right**).

Figure 9 and Figure 10 illustrate binary depth maps from our radial motion experiments that show the locations of zero-value pixels (in black) and non-zero value pixels (in white). Figure 9 corresponds to the results when the Siemens Star is parallel to the image plane of the ToF sensor. Figure 10 corresponds to the results when the Siemens Star is at a 30° rotation from the image plane of the ToF sensor. In each figure, the first row shows depth maps of the Siemens Star in a static position without motion, the middle row shows depth maps with synthetic radial motion blur at different speeds, and the bottom row shows depth maps with real motion blur at different speeds.

Our results show that in general as motion blur occurs, the number of zero-value pixels that occur near the edges of the fan blades increases. As the fan blades move, the motion of the fan blades causes pixels near the edges of the fan blades to become zero-value pixels or flying pixels that are no longer on the surface of the fan blade. As the speed of the Siemens Star increases, the amount of motion blur increases which also increases the number of zero-value pixels and flying pixels that are present near the edges of the fan blades in the depth map. Our results also show that this behavior results in the surface area of the fan blades appearing reduced due to an increase in the number of pixels on the surface of the fan blades becoming zero-value pixels and flying pixels.

As the rotation angle between the Siemens Star and the image plane of the ToF sensor increases, the number of zero-value pixels in the portion of the Siemens Star that is furthest away from the ToF sensor begins to decrease and the number of zero-value pixels in the portion of the Siemens Star that is closer to the ToF sensor begins to increase. This behavior can be seen in Figure 10. As the rotation angle between the Siemens Star and the image plane of the ToF sensor approaches 45°, zero-values pixels become harder to detect in the portion of the Siemens Star that is furthest away from the ToF sensor because the IR light that is projected by the ToF sensor begins to reflect away from the ToF sensor.

Table 4, Table 5 and Table 6 show the performance of the proposed synthetically generated radial motion blur process. For each speed, we compare the depth maps of the Siemens Star with synthetic motion blur and real motion blur. Our experiments were performed using a user-defined constant height value of one pixel, two pixels, and three pixels for the ROI orthogonal dimension.

Table 4 shows that as the speed of the Siemens Star increases, the BF score decreases. Table 4 also shows that as the rotation angle between the Siemens Star and the image plane of the ToF sensor increases, the standard deviation of the BF scores increases as the speed of the Siemens Star increases. The BF score also begins to degrade as the ROI orthogonal dimension increases. Our results show that our framework for synthetically generating radial motion blur is able to achieve an average BF score of 0.7192 over a range of speeds between 60 RPM and 135 RPM and over a range of rotation angles between 0° and 30°.

Table 5 shows that as the speed of the Siemens Star increases, the RMSE increases. Table 5 also shows that as the rotation angle between the Siemens Star and the image plane of the ToF sensor increases, the standard deviation of the RMSE increases as the speed of the Siemens Star increases. The RMSE also increases as the ROI orthogonal dimension increases. Table 6 shows the same behavior for the RMSE-R as the RMSE. Our results show that our framework for synthetically generating radial motion blur is able to achieve an average RMSE of 23.8914 and an average RMSE-R of 0.0241 over a range of speeds between 60 RPM ad 135 RPM and over a range of rotation angles between 0° and 30°.

In general, our results show that the locations of zero value pixels can be accurately predicted for radial motion which translates to a reasonably accurate synthetic representation of radial motion blur.

### 3.5. Linear Motion Blur Experimental Results

**Figure 11:**
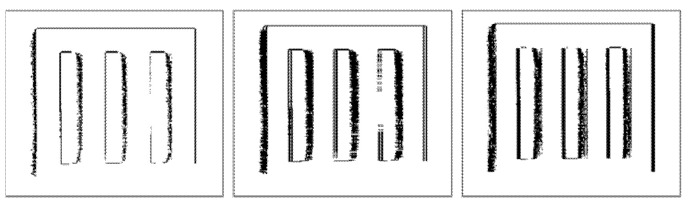
Linear motion parallel to the image plane of the ToF sensor without motion (**left**), with synthetic motion blur (**middle**), and with real linear motion blur (**right**).

**Figure 12:**
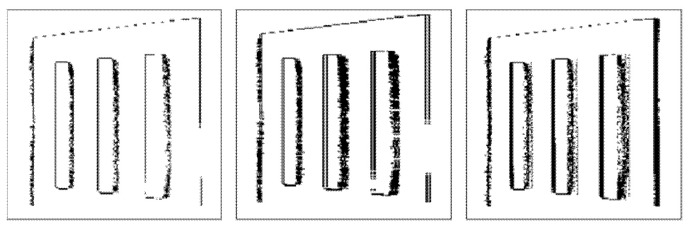
Linear motion with a 30° rotation from the image plane of the ToF sensor without motion (**left**), with synthetic motion blur (**middle**), and with real linear motion blur (**right**).

Figure 11 and Figure 12 illustrate binary depth maps from our linear motion experiments. Figure 11 corresponds to the results when the linear target is parallel to the image plane of the ToF sensor. Figure 12 corresponds to the results when the linear target is configured at a 30° rotation from the image plane of the ToF sensor.

Similar to our findings in our radial motion experiments, our results show that as the rotation angle between the linear target and the image plane of the ToF sensor increases, the number of zero-value pixels in the portion of the linear target that is furthest away from the ToF sensor begins to decrease and the number of zero-value pixels in the portion of the linear target that is closer to the ToF sensor begins to increase. This behavior can be seen in Figure 12. Increasing the ROI orthogonal dimension also negatively impacts the BF score, the RMSE, and the RMSE-R, as shown in Table 7. Our results demonstrate that our framework for synthetically generating linear motion blur is able to achieve an average BF score of 0.8793, an average RMSE of 7.4633, and an average RMSE ratio of 0.0073 over a range of rotation angles between 0° and 30°.

Our results show the locations of zero value pixels can also be accurately predicted for linear motion which enables a reasonably accurate synthetic representation of linear motion blur.

### 3.6. Combined Radial and Linear Motion Blur Experimental Results

**Figure 13:**
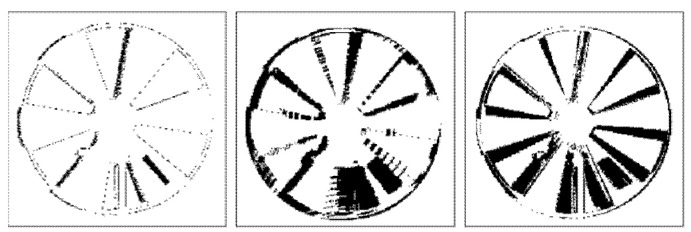
Combined radial-linear motion from left to right without motion (**left**), with synthetic motion blur (**middle**), and with real linear motion blur (**right**).

**Figure 14:**
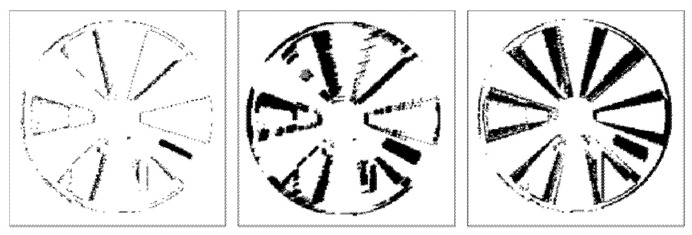
Combined radial-linear motion from right to left without motion (**left**), with synthetic motion blur (**middle**), and with real linear motion blur (**right**).

Figure 13 and Figure 14 illustrate binary depth maps from our combined radial-linear motion experiments. Figure 13 corresponds to the results when the target is rotating clockwise while moving from left to right. Figure 14 corresponds to the results when the target is rotating while moving from right to left.

In our experiments for combined radial-linear motion, one of the fan blades of the Siemens Star is marked with an IR reflective marker that artificially creates a hole in the fan blade. Our results show that as motion blur occurs, the number of zero-value pixels that are present in the IR reflective marker and near the edges of the fan blades increases.

Our results show how the direction of the radial motion combined with the direction of the linear motion affects the number of zero-value pixels that are present. When the radial motion is in the same direction as the linear motion, there is an increase in the number of zero-value pixels that are present. When the radial motion is in the opposite direction from the linear motion, there is a decrease in the number of zero-value pixels that are present. This behavior can be seen in the upper and lower hemispheres of the Siemens Star in Figure 13 and Figure 14. Since there is no rotation angle between the target and the image plane of the ToF sensor, there is no significant difference in the number of zero-value pixels that are present at the left and right edges of the target.

As shown in Table 8, the proposed framework for synthetically generating combined radial-linear motion blur achieves an average BF score of 0.6201, an average RMSE of 99.6939, and an average RMSE ratio of 0.0694 while operating the target with a radial speed of 135 RPM and a linear speed of 3.3647 m/s.

### 3.7. Validating Synthetic Motion Generating Framework Using Other ToF Sensors

**Figure 15:**
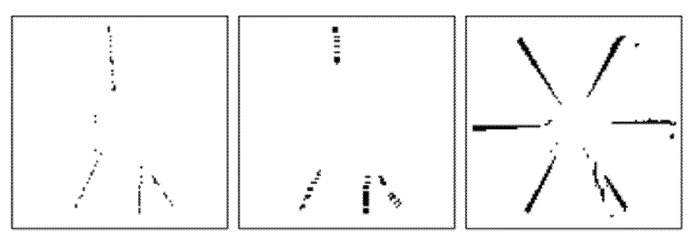
Siemens Star that is parallel to the image plane of the TI OPT8241 ToF sensor without motion (**left**), with synthetic motion blur (**middle**), with real motion blur (**right**).

Since the presence of invalid pixels due to motion blur is an artifact of the underlying hardware architecture that all ToF sensors use to determine depth values [19,31,32,33,34], this means that our framework can be generally applied to depth maps from other ToF sensors. To verify that our framework can be successfully used with other ToF sensors, we applied our synthetic motion blur generating process to a depth map that was obtained from a TI OPT8241 ToF sensor [54]. Figure 15 illustrates an example of the experiment results using our proposed framework with the TI OPT8241 ToF sensor. In the example shown in Figure 15, the Siemens Star is parallel to the image plane of the TI OPT8241 ToF sensor and is rotating at a speed of 135 RPM.

Note that the TI OPT8241 ToF sensor uses internal filtering, which we cannot disable, to remove zero-value pixels in both the static depth map and the depth map with real motion blur. This filtering causes randomness in locations of zero-value pixels and in which depth discontinuities they appear. For example, the zero-value pixels along some edges of the Siemens Star in the static depth map are filtered out by the ToF sensor when generating the depth map with real motion blur. Similarly, the zero-value pixels along some edges of the Siemens Star in the depth map with real motion blur are filtered out by the ToF sensor when generating the static depth map. Our framework relies on the presence of zero-value pixels within the static depth map to create additional zero-value pixels in the depth map with synthetic motion blur. For this reason, the zero-value pixels for some of the edges in the depth map with real motion blur are not represented in the depth map with synthetic motion blur. As shown in this example, our framework can accurately predict the locations of zero value pixels to generate synthetic motion blur. For conciseness, only one example is provided, however, similar results were obtained for other types of motion and orientations.

## 4. Conclusions

In this work, we present a framework for synthetically generating motion blur in depth maps that mimics the behavior of real motion blur that is observed using a ToF sensor. This work introduces an improved probabilistic model that can predict the location of zero-value pixels that are present when motion blur occurs and there is a rotation angle between an object and the image plane of a ToF sensor. This work also introduces a process for synthetically generating combined radial-linear motion blur.

One of the limitations in our framework is that our framework relies on the presence of zero-value pixels in the static depth map to create new additional zero-value pixels in our depth map with synthetic motion blur. Future work can work to incorporate more sophisticated models to generate zero-value pixels for multiple object planes. A more comprehensive methodology can also be investigated for accounting for perspective distortion as the rotation angle between an object and the image plane of a ToF sensor increases.

## Figures and Tables

**Table 1:** Frequently used notations in this paper.

Symbol	Description
*p*(*x*, *y*)	The pixel of a depth map in Cartesian coordinates
*d*	The distance between the image plane of a ToF sensor and a point in the object plane
𝜃	The rotation angle between the motion of an object and the image plane of a ToF sensor
***I***	The initial depth map in 2D matrix of pixels with dimensions M × N
***I**_**b**_*	The binary depth map in 2D matrix of pixels with dimensions M × N
***I**_**f**_*	The blurred depth map in 2D matrix of pixels with dimensions M × N
$\vec{v}$	The velocity of a pixel at *p*(*x*, *y*)
*v^x^*	The velocity component in the x-direction
*v^y^*	The velocity component in the y-direction
*w*	The ROI dimension tangential to the velocity of a pixel at *p*(*x*, *y*)
*h*	The ROI dimension orthogonal to the velocity of a pixel at *p*(*x*, *y*)
*s*(*x*, *y*)	The perspective distortion scaling function of a pixel at *p*(*x*, *y*)
*β*	The rotation angle of an ROI within a depth map
$\vec{\varphi }$	The angular motor velocity of a radial motion
$\vec{\mu }$	The linear motor velocity of a linear motion
{*n*_0_, *n_1_*, …, *n_l_*}	The set of neighboring pixels within an ROI for a given pixel *p*(*x*, *y*)
*p_p_*	The initial probability of a given pixel *p*(*x*, *y*) having a value of zero
*ε*(*s_p_*, *s_ni_*)	The potential function of the *i*th neighboring pixel to the given pixel *p*(*x*, *y*)
${\tilde{p}}_{p}$	The predicted probability of a given pixel *p*(*x*, *y*) having a value of zero
${\tilde{s}}_{p}$	The predicted state of a given pixel *p*(*x*, *y*) having a value of one or zero in a binary depth map

**Table 2:** Ratio of zero-value pixels in depth maps with real motion.

	Angle between the Sensor Plane and the Plane of Rotation of the Siemens Star			
Speed	0	15	20	30
60 RPM	0.1212	0.1140	0.1179	0.1170
100 RPM	0.1884	0.1779	0.1789	0.1739
135 RPM	0.2483	0.2355	0.2325	0.2261

**Table 3:** Percentage of zero-value pixels from a static depth map that are present in a depth map with real motion blur.

	Angle between the Sensor Plane and the Plane of Rotation of the Siemens Star			
Speed	0	15	20	30
60 RPM	77.55%	73.29%	69.18%	72.34%
100 RPM	74.45%	75.07%	70.28%	72.22%
135 RPM	81.80%	79.95%	75.52%	77.84%

**Table 4:** Synthetic radial motion blur performance-BF score.

Height Value: 1 Pixel	Rotation Angle			
Speed	0	15	20	30
60 RPM	0.7873	0.7802	0.7866	0.7872
100 RPM	0.7157	0.7091	0.7211	0.7337
135 RPM	0.6401	0.6362	0.6593	0.6740
Height value: 2 pixels	Rotation Angle			
Speed	0	15	20	30
60 RPM	0.7757	0.7598	0.7579	0.7688
100 RPM	0.6633	0.6604	0.6745	0.6991
135 RPM	0.5808	0.5803	0.6075	0.6334
Height value: 3 pixels	Rotation Angle			
Speed	0	15	20	30
60 RPM	0.0170	0.0188	0.0229	0.0230
100 RPM	0.0220	0.0248	0.0285	0.0282
135 RPM	0.0312	0.0330	0.0389	0.0378

**Table 5:** Synthetic radial motion blur performance-RMSE (mm).

Height value: 1 pixel	Rotation Angle			
Speed	0	15	20	30
60 RPM	15.5832	16.3344	20.3076	19.7015
100 RPM	19.06	21.3124	24.9468	24.7282
135 RPM	27.5602	28.9867	35.3897	32.7857
Height value: 2 pixels	Rotation Angle			
Speed	0	15	20	30
60 RPM	16.5201	16.4827	19.6956	19.7207
100 RPM	20.6372	22.3107	25.4945	25.3598
135 RPM	28.6869	30.2006	35.8323	34.1595
Height value: 3 pixels	Rotation Angle			
Speed	0	15	20	30
60 RPM	16.5795	18.1961	22.7809	22.9275
100 RPM	21.6180	24.0258	28.1812	28.1346
135 RPM	30.6426	31.9231	38.7674	37.3976

**Table 6:** Synthetic radial motion blur performance-RMSE-R.

Height Value: 1 Pixel	Rotation Angle			
Speed	0	15	20	30
60 RPM	0.0159	0.0168	0.02	0.0194
100 RPM	0.0195	0.0217	0.0251	0.0245
135 RPM	0.0281	0.0295	0.0354	0.0327
Height value: 2 pixels	Rotation Angle			
Speed	0	15	20	30
60 RPM	0.0170	0.0172	0.0202	0.0199
100 RPM	0.0208	0.0230	0.0257	0.0255
135 RPM	0.0291	0.0310	0.0358	0.0343
Height value: 3 pixels	Rotation Angle			
Speed	0	15	20	30
60 RPM	0.0170	0.0188	0.0229	0.0230
100 RPM	0.0220	0.0248	0.0285	0.0282
135 RPM	0.0312	0.0330	0.0389	0.0378

**Table 7:** Synthetic linear motion blur performance.

Height Value: 1 Pixel	Rotation Angle			
Metrics	0	15	20	30
BF Score	0.8943	0.8498	0.8954	0.8778
RMSE (mm)	8.2291	7.4568	7.2266	6.9405
RMSE-R	0.0082	0.0072	0.0072	0.0066
Height value: 2 pixels	Rotation Angle			
Metrics	0	15	20	30
BF Score	0.8292	0.7163	0.8493	0.8074
RMSE (mm)	8.2540	9.1342	8.0566	7.7380
RMSE-R	0.0082	0.0086	0.0081	0.0076
Height value: 3 pixels	Rotation Angle			
Metrics	0	15	20	30
BF Score	0.8191	0.6592	0.8127	0.7752
RMSE (mm)	8.7983	9.3763	8.5384	8.1487
RMSE-R	0.0088	0.0089	0.0085	0.0081

**Table 8:** Synthetic combined radial and linear motion blur performance.

Height Value: 1 Pixel	Movement Direction	
Metrics	Left-to-Right	Right-to-Left
BF Score	0.6092	0.6309
RMSE (mm)	106.0410	93.3467
RMSE-R	0.0733	0.0654
Height value: 2 pixels	Movement Direction	
Metrics	Left-to-Right	Right-to-Left
BF Score	0.5943	0.6131
RMSE (mm)	153.2062	148.2040
RMSE-R	0.0994	0.0965
Height value: 3 pixels	Movement Direction	
Metrics	Left-to-Right	Right-to-Left
BF Score	0.5790	0.6013
RMSE (mm)	197.8745	202.5617
RMSE-R	0.1254	0.1281

## Data Availability

Not applicable.
